# The Antioxidant Activity and Oxidative Stability of Cold-Pressed Oils

**DOI:** 10.1007/s11746-014-2479-1

**Published:** 2014-05-16

**Authors:** Anna Prescha, Magdalena Grajzer, Martyna Dedyk, Halina Grajeta

**Affiliations:** Department of Food Science and Dietetics, Wroclaw Medical University, Borowska 211, 50-556 Wrocław, Poland

**Keywords:** Oxidative stability, DPPH, Shelf life, Cold-pressed oils

## Abstract

In our study, we characterized the antioxidant activity and oxidative stability of cold-pressed macadamia, avocado, sesame, safflower, pumpkin, rose hip, Linola, flaxseed, walnut, hempseed, poppy, and milk thistle oils. The radical scavenging activity of the non-fractionated fresh oil, as well as the lipophilic and hydrophilic fractions of the oil was determined using a 1,1-diphenyl-2-picrylhydrazyl (DPPH) assay. The fatty acid composition of the fresh and stored oils was analyzed by gas chromatography. The acid value, peroxide value, *p*-anisidine value and conjugated diene and triene contents in the fresh oils, as well as in those stored throughout the whole period of their shelf life, were measured by CEN ISO methods. The antioxidant activity of the oils expressed as Trolox equivalent antioxidant capacity (TEAC), ranged from 0.17 to 2.32 mM. The lipophilic fractions of the oils were characterized by much higher antioxidant activity than the hydrophilic ones. There were no significant changes in fatty acid composition and only slight changes in the oxidative stability parameters of the oils during their shelf life. Through the assessment of the relationship between antiradical activity and the oxidative stability of oils, it is proposed that a DPPH assay predicts the formation of oxidation products in cold-pressed oils—however, the correlations differ in fractionated and nonfractionated oils.

## Introduction

In recent years, a number of cold-pressed oils from the seeds and fruits of different plants have appeared on the Polish market. These oils have specific characteristics and flavors, and often contain valuable bioactive substances. Apart from valuable unsaturated fatty acids, these oils contain more natural antioxidants, such as tocopherols and phenolic compounds, than their refined counterparts [1–3]. Cold-pressed oils have long shelf life stability due to the presence of antioxidants and other molecules that stabilize the oil with respect to auto-oxidation. The stability of cold-pressed oils is usually set for 6 or 12 months and generally limited by the content of polyunsaturated fatty acids (PUFA), especially alpha-linolenic acid, and the quantity of antioxidants [4]. The high content of PUFA and other substances favoring oil decomposition at high temperature restrains the use of unrefined oils for frying, since frying negatively affects the consumer acceptability of finished products in terms of color, flavor, etc. [5]. As far as this issue is concerned, refined oils have an advantage over unrefined ones. However, the organoleptic properties and health benefits of cold-pressed oils, which result from the content of natural minor components, are increasingly valued by consumers. Therefore, studies on the factors that can influence the quality of these oils are essential.

The oxidative processes that may occur during the shelf life of cold-pressed oils do not result in an increase in oxidative stability parameters above the adopted limits [6]. However, they could affect the stability of oils during storage in domestic conditions, during which they are exposed to light, kept open causing contact with the air, or kept at an ambient temperature [7]. Conjugated dienoic and trienoic acids, as well as peroxide, acid and *p*-anisidine values, are commonly used to measure oxidative stability in cold-pressed oils [6, 7]. Resistance to oxidative changes in oils can also be assessed using a DPPH assay. This method is based on a single electron transfer mechanism and measures the ability of the antioxidants in oil to reduce a stable DPPH radical [8]. The DPPH assay has been adopted for the determination of the antiradical activity of the hydrophilic or lipophilic antioxidants of oil, as well as for the total antioxidant activity of nonfractionated oil [9]. The DPPH assay has been shown to be a good predictor of the oxidative stability of oils as determined using the Rancimat test and other thermal oxidations of oil [10, 11]. These stability tests require elevated temperatures of oxidation (even 100 °C and higher) and exogenous oxidation promoters, which are not relevant to the normal storage conditions of cold-pressed oils [10].

Therefore, the objective of this research was to assess the initial antioxidant activity and oxidative stability of selected cold pressed oils throughout their shelf life as well as to characterize the relationship between the antioxidant activity of nonfractionated and fractionated oils and the parameters of oil oxidation measured.

## Materials and Methods

Sample details of the 12 kinds of cold-pressed oils used in the study are provided in Table 1. The oils used in the research were purchased fresh (within 4 weeks of manufacture) from local grocery stores in Wroclaw (Poland) or provided fresh by Oleofarm (Pietrzykowice, Poland), a manufacturer of edible cold-pressed oils. The analyses were conducted on fresh oils as planned, subsequently after 3 and 6 months of storage, and, in the case of five of the oils, also after 12 months. The oils were stored in their original glass bottles at a temperature of 20 °C and in a 12/12 h light/dark regime. Each sample analysis was replicated at least three times to ensure overall accuracy at a minimum of 5 % of CV (coefficient of variation).

**Table 1:** The names of the cold-pressed oils, country of origin and shelf life

Oil type	Abbreviated name	*n*	Shelf life (months)	Country of origin
Macadamia oil	MACO	3	12	South Africa (*n* = 3)
Avocado oil	AVO	4	12	South Africa (*n* = 3) Poland (*n* = 1)
Sesame oil	SESO	3	12	Poland (*n* = 3)
Safflower oil	SAFO	5	12	Poland (*n* = 3) France (*n* = 2)
Pumpkin oil	PUMO	3	12	Poland (*n* = 2) Austria (*n* = 1)
Rose hip oil	ROSO	4	6	Poland (*n* = 4)
Linola oil	LINO	7	6	Poland (*n* = 7)
Flaxseed oil	FLAO	4	6	Poland (*n* = 3) Italy (*n* = 1)
Walnut oil	WALO	4	6	Poland (*n* = 3) Austria (*n* = 1)
Hempseed oil	HEMO	4	6	Poland (*n* = 2) France (*n* = 2)
Poppy oil	POPO	4	6	Poland (*n* = 3) France (*n* = 1)
Milk thistle oil	MILO	3	6	Poland (*n* = 2) Czech Republic (*n* = 1)

*n* number of brands of oil

### Radical Scavenging Activity

To evaluate the antioxidant activity of the oils, spectrophotometric analysis was performed using 1,1-diphenyl-2-picrylhydrazyl (DPPH) [12]. The DPPH assay was used to determine the antioxidant activity in nonfractionated oil, and in hydrophilic (HF) and lipophilic fractions (LF). To separate the HF and LF, 500 µl of oil was mixed with 500 µl of methanol, and then centrifuged to allow the fractions to separate. Spectrophotometric readings were carried out after a 1 h period of incubation with a Genesys 6 Thermo spectrophotometer at 517 nm using a 10-mm quartz cuvette. The data were expressed as a Trolox equivalent antioxidant capacity (TEAC, mM/kg) using a Trolox calibration curve in the range 0.02–4.00 mM.

### Fatty Acid Composition

Fatty acid methyl esters (FAME) were prepared employing the method developed by Prescha et al. [13]. Analysis of FAME was performed with gas chromatograph 6890 N (Agilent Technologies, USA) equipped with a FID detector and a capillary column CP-SIL88 50 m × 0.25 mm × 0.5 μm (Varian, USA). Hydrogen was used as the carrier gas at a flow rate of 1.5 ml/min and the separation was carried out at a temperature set from 110 °C (for 5 min) to 220 °C; the temperature being increased at a rate of 2 °C/min. The identification of particular fatty acids was accomplished by a comparison with external standards. Pentadecanoic acid was used as an internal standard for quantitative analysis and Chemstation v. B.04.02 was used to calculate the results.

Acid value (AV), peroxide value (PV), *p*-anisidine value (*p*-AV), conjugated dienes [14] and trienes (CT).

The acid, peroxide and *p*-anisidine values were determined in accordance with CEN ISO 660:2009 [15], CEN ISO 3960:2010 [16] and CEN ISO 6885:2008 [17], respectively. Spectrophotometric determination of the CD and CT of the cold-pressed oils was performed in accordance with CEN ISO 3656:2002 [18].

### Statistical Analysis

An analysis of variance was carried out and followed by Tukey’s *post hoc* test for intergroup comparison of parametric data. When dealing with nonparametric data, the Kruskal–Wallis test was performed. Differences were considered statistically significant at *p* < 0.05. The correlations of data were assessed using the Spearman rank correlation test. Data were evaluated by the Statistica 10.0 software package (StatSoft Poland).

## Results and Discussion

### DPPH Radical Scavenging Activity of Cold-Pressed Oils

Table 2 shows the DPPH antioxidant activity of cold-pressed oils expressed in TEAC, for both hydrophilic and lipophilic fractions, as well as for oils not subjected to extraction. The DPPH radical scavenging activity of the studied oils ranged from 0.17 up to 2.32 mM TEAC/kg. The lipophilic fraction of all the studied oils was characterized by much higher activity than its hydrophilic equivalent, which is corroborative with previous studies and reflects more significant amounts of lipophilic antioxidants (tocopherols) than hydrophilic ones (phenolic compounds) present in oils [8]. The ratio LF/HF ranged from 1.31 (MACO) up to 7.60 (SAFO). Apart from SAFO, LINO and FLAO were also characterized by high LF/HF ratios (7.37 and 7.06, respectively). A similar result was obtained, for FLAO only, by Tuberoso et al. [12]. In individual samples of SESO and WALO, high LF/HF ratios were observed once more (9.36 and 7.34, respectively). ROSO was characterized by the largest antioxidant activity (2.32 mM TEAC/kg), which may result from the high content or synergistic activity of antioxidant compounds in this oil. Data on antioxidants occurring in ROSO is extremely sparse, however, it has been found that ROSO contains considerable amounts of carotenoids (46–145 mg/kg) [19].

**Table 2:** The results of antiradical scavenging activity of oils–DPPH assay (mM TAEC/kg)

Oil type	Mean ± SD^a^ (range)			
	Oil	LF	HF	LF/HF
MACO	0.17 ± 0.03 (0.14–0.20)	0.12 ± 0.06 (0.05–0.16)	0.09 ± 0.07 (0.04–0.16)	1.31 ± 0.02 (0.81–4.05)
AVO	0.58 ± 0.08 (0.52–0.66)	0.51 ± 0.11 (0.40–0.64)	0.09 ± 0.03 (0.07–0.12)	5.74 ± 0.30 (4.95–7.26)
SESO	1.38 ± 0.47 (0.96–1.94)	1.15 ± 0.34 (0.80–1.58)	0.50 ± 0.34 (0.26–1.05)	2.33 ± 0.47 (1.08–9.36)
SAFO	1.77 ± 0.05 (1.74–1.83)	1.57 ± 0.04 (1.54–1.63)	0.21 ± 0.03 (0.18–0.25)	7.60 ± 0.97 (6.17–9.04)
PUMO	1.44 ± 0.33 (1.11–1.77)	1.35 ± 0.19 (1.17–1.54)	0.41 ± 0.22 (0.23–0.65)	3.30 ± 0.66 (2.38–3.38)
ROSO	2.32 ± 0.04 (2.28–2.37)	2.14 ± 0.13 (2.05–2.30)	0.39 ± 0.03 (0.36–0.42)	5.49 ± 1.24 (4.97–6.30)
LINO	1.68 ± 0.21 (1.52–1.92)	1.52 ± 0.24 (1.33–1.80)	0.21 ± 0.03 (0.18–0.23)	7.37 ± 0.93 (6.14–10.00)
FLAO	1.58 ± 0.17 (1.30–1.72)	1.35 ± 0.21 (1.07–1.66)	0.19 ± 0.03 (0.14–23)	7.06 ± 0.82 (5.66–10.04)
WALO	1.28 ± 0.12 (1.15–1.37)	1.08 ± 0.09 (1.02–1.18)	0.37 ± 0.29 (0.14–0.69)	2.93 ± 0.50 (1.70–7.34)
HEMO	1.74 ± 0.26 (1.47–2.00)	1.51 ± 0.23 (1.25–1.66)	0.35 ± 0.06 (0.29–0.42)	4.27 ± 0.82 (3.85–4.75)
POPO	0.72 ± 0.08 (0.67–0.81)	0.67 ± 0.13 (0.55–0.81)	0.22 ± 0.17 (0.12–0.41)	3.03 ± 0.32 (1.53–6.37)
MILO	1.70 ± 0.23 (1.56–1.97)	1.28 ± 0.16 (1.12–1.44)	0.28 ± 0.06 (0.24–0.35)	4.57 ± 0.71 (4.17–4.98)

^a^Mean and standard deviations (SD) values were obtained from analyses of brand set of one oil type

### Fatty Acid Composition

Table 3 shows the fatty acid composition of the fresh oils studied. The highest content of C18:2n-6 was found in SAFO, LINO and POPO, of C18:3n-3 in FLAO and of C18:1n-9 in MACO and AVO. MACO contained the highest amounts of C16:1n-7 among the studied oils, and HEMO was distinguished by approximately 2 % of C18:3n-6. Fatty acid composition in oils was in agreement with the previous data [12, 20–24]. However, the main fatty acid content in SAFO was found to be outside the limit of the range presented in the literature: 20.6 %o C18:1n-9 (typical content 11–16 %) and 67.3 % of C18:2n-6 (typical content 72–79 %) [25, 26]. Table 4 presents SFA, MUFA, PUFA and TFA content in fresh and stored oils. A large variability of MUFA and PUFA contents between the analyzed brands of SAFO, PUMO, FLAO and HEMO was observed (relative standard deviation was up to 33.3 % for MUFA in HEMO). Such variability of fatty acid composition of these oils can be found in literature [12, 20, 27–31]. ROSO was characterized by the lowest SFA and highest PUFA contents (an especially high percentage of alpha-linolenic acid), suggesting its susceptibility to oxidation. However, no significant changes in fatty acid contents in ROSO, or other studied oils, were found during storage. In the correlation test we also observed the effect of the antiradical activity of oils on inhibition of PUFA deterioration, expressed as percentage of change of PUFA content after 12 months of storage (Table 5). The TFA content was very low in fresh oils (0.1–0.7 %) and only a slight increase could be observed during storage, as the highest value did not exceed 0.13 % at the end of their shelf life. From the correlation test we can conclude that the antioxidants of oils could protect from *trans* isomerization of fatty acids for up to 6 months of storage.

**Table 3:** The fatty acids compositions of studied oils

Fatty acids (%, mean ± SD)^a^												
Oil type	C14:0	C16:0	C16:1n-7	C17	C18:0	C18:1n-9	C18:2n-6	C18:3n-3	C18:3n-6	C20	C20:1n-9	other
MACO	0.8 ± 0.1	8.2 ± 0.6	18.1 ± 1.1	0.1 ± 0.0	3.3 ± 0.3	58.9 ± 2.0	3.5 ± 0.9	0.5 ± 0.5	ND	2.7 ± 0.1	2.4 ± 0.1	2.4
AVO	0.1 ± 0.1	17.5 ± 1.0	8.1 ± 0.9	0.6 ± 0.9	0.7 ± 0.2	61.0 ± 1.5	10.5 ± 0.7	0.8 ± 0.2	ND	0.1 ± 0.1	0.3 ± 0.1	0.3
SESO	0.1 ± 0.0	10.4 ± 1.3	0.2 ± 0.1	t	5.3 ± 0.8	39.5 ± 0.9	42.9 ± 1.8	0.5 ± 0.2	ND	0.5 ± 0.1	0.2 ± 0.0	0.8
SAFO	0.1 ± 0.0	6.2 ± 0.4	0.1 ± 0.0	t	2.88 ± 0.5	20.6 ± 4.8	67.3 ± 3.0	0.6 ± 0.2	ND	0.8 ± 0.3	0.3 ± 0.0	1.0
PUMO	0.2 ± 0.0	12.3 ± 0.9	0.1 ± 0.0	t	6.1 ± 0.4	32.3 ± 6.2	47.2 ± 5.5	0.4 ± 0.1	ND	0.4 ± 0.0	0.1 ± 0.0	0.3
ROSO	0.1 ± 0.0	3.8 ± 0.4	ND	t	1.8 ± 0.1	14.6 ± 0.2	44.1 ± 0.3	34.0 ± 0.4	0.1 ± 0.1	0.6 ± 0.3	0.3 ± 0.2	0.4
LINO	0.1 ± 0.0	5.6 ± 0.6	ND	t	3.9 ± 0.1	16.6 ± 0.7	71.05 ± 0.4	2.0 ± 0.2	ND	0.1 ± 0.0	0.1 ± 0.0	0.4
FLAO	0.1 ± 0.0	5.2 ± 0.2	ND	t	4.2 ± 0.5	19.3 ± 3.0	14.0 ± 1.4	51.2 ± 3.7	ND	0.2 ± 0.0	0.1 ± 0.1	0.6
WALO	t	7.1 ± 0.3	0.1 ± 0.0	ND	2.6 ± 0.1	18.4 ± 1.2	59.7 ± 1.9	11.2 ± 0.7	ND	0.1 ± 0.0	0.5 ± 0.3	0.1
HEMO	0.1 ± 0.0	6.1 ± 0.2	ND	t	2.6 ± 0.3	12.5 ± 4.2	55.2 ± 1.5	18.9 ± 2.6	2.2 ± 0.5	0.7 ± 0.2	0.4 ± 0.1	1.0
POPO	0.1 ± 0.0	9.6 ± 0.5	0.2 ± 0.0	t	2.3 ± 0.1	15.2 ± 0.4	71.00 ± 0.6	0.8 ± 0.2	ND	0.1 ± 0.0	0.6 ± 0.3	0.1
MILO	0.2 ± 0.1	8.2 ± 0.4	0.1 ± 0.0	t	4.8 ± 0.4	22.8 ± 0.8	55.8 ± 1.6	0.3 ± 0.0	ND	2.9 ± 0.2	0.9 ± 0.1	4.1

^a^Weight percent of total fatty acids, mean and standard deviation (SD) values obtained from analyses of brand set of one oil type

*ND* not detected, *t* trace <0.05 %

**Table 4:** The fatty acid groups in fresh oils and during storage

Oil type	Fatty acid group (%, mean value ± SD)^b^															
	MUFA				PUFA				SFA				TFA			
	Months of storage				Months of storage				Months of storage				Months of storage			
	0	3	6	12	0	3	6	12	0	3	6	12	0	3	6	12
MACO	79.9 ± 0.8^a^	79.4 ± 0.5^a^	80.0 ± 0.6^a^	79.1 ± 0.9^a^	4.0 ± 1.5^a^	4.1 ± 1.2^a^	3.8 ± 1.4^a^	4.5 ± 1.6^a^	15.4 ± 0.5^a^	16.1 ± 0.5^a^	16.4 ± 0.7^a^	16.3 ± 0.2^a^	0.01 ± 0.01^a^	0.06 ± 0.03^a^	0.08 ± 0.05^a^	0.07 ± 0.05^a^
AVO	69.4 ± 1.1^a^	69.9 ± 0.6^a^	70.5 ± 1.0^a^	69.8 ± 0.6^a^	11.0 ± 0.8^a^	11.4 ± 1.0^a^	11.7 ± 0.9^a^	10.8 ± 1.0^a^	18.7 ± 0.9^a^	19.2 ± 0.6^a^	18.5 ± 0.6^a^	18.9 ± 0.7^a^	0.11 ± 0.06^a^	0.04 ± 0.02^a^	0.06 ± 0.02^a^	0.03 ± 0.03^a^
SESO	39.8 ± 0.8^a^	40.0 ± 0.9^a^	40.2 ± 0.7^a^	40.5 ± 1.1^a^	43.4 ± 1.0^a^	44.1 ± 1.2^a^	43.6 ± 1.3^a^	42.7 ± 1.6^a^	16.7 ± 1.8^a^	15.8 ± 0.5^a^	16.1 ± 0.7^a^	16.7 ± 0.8^a^	0.07 ± 0.04^a^	0.05 ± 0.03^a^	0.10 ± 0.05^a^	0.13 ± 0.05^a^
SAFO	21.2 ± 4.7^a^	21.3 ± 4.6^a^	21.3 ± 4.9^a^	21.2 ± 4.5^a^	67.9 ± 3.2^a^	67.7 ± 3.1^a^	67.5 ± 3.2^a^	67.7 ± 2.9^a^	10.9 ± 1.5^a^	11.0 ± 1.6^a^	11.2 ± 1.5^a^	11.0 ± 1.7^a^	0.02 ± 0.02^a^	0.04 ± 0.03^a^	0.03 ± 0.02^a^	0.07 ± 0.03^a^
PUMO	33.0 ± 6.2^a^	33.6 ± 6.2^a^	33.6 ± 6.2^a^	32.7 ± 7.2^a^	47.6 ± 5.6^a^	47.3 ± 5.1^a^	46.5 ± 5.1^a^	46.6 ± 5.5^a^	19.4 ± 0.9^a^	19.1 ± 0.8^a^	19.9 ± 0.8^a^	20.7 ± 1.5^a^	0.01 ± 0.01^a^	0.07 ± 0.04^a^	0.06 ± 0.04^a^	0.07 ± 0.04^a^
ROSO	15.2 ± 0.5^a^	15.5 ± 0.5^a^	15.2 ± 0.6^a^	–	78.4 ± 0.7^a^	77.9 ± 0.6^a^	77.6 ± 0.5^a^	–	6.5 ± 0.4^a^	6.7 ± 0.2^a^	7.3 ± 0.8^a^	–	0.03 ± 0.01^a^	0.03 ± 0.01^a^	0.05 ± 0.03^a^	–
LINO	16.9 ± 0.7^a^	17.5 ± 0.9^a^	16.4 ± 0.8^a^	–	73.0 ± 0.2^a^	72.3 ± 0.2^a^	71.9 ± 0.4^a^	–	10.0 ± 0.6^a^	10.1 ± 0.8^a^	11.1 ± 1.1^a^	–	0.00 ± 0.00^a^	0.04 ± 0.02^a^	0.04 ± 0.02^a^	–
FLAO	20.6 ± 3.2^a^	20.5 ± 3.1^a^	20.6 ± 3.3^a^	–	68.8 ± 3.0^a^	69.1 ± 3.0^a^	68.8 ± 3.4^a^	–	10.4 ± 0.4^a^	10.4 ± 0.4^a^	10.6 ± 0.5^a^	–	0.02 ± 0.01^a^	0.03 ± 0.01^a^	0.05 ± 0.03^a^	–
WALO	19.1 ± 0.5^a^	19.4 ± 0.9^a^	19.2 ± 0.9^a^	–	70.9 ± 0.2^a^	70.5 ± 0.9^a^	70.0 ± 1.3^a^	–	10.0 ± 0.4^a^	10.2 ± 0.2^a^	10.7 ± 0.2^a^	–	0.03 ± 0.03^a^	0.05 ± 0.02^a^	0.06 ± 0.02^a^	–
HEMO	13.1 ± 4.1^a^	13.2 ± 4.2^a^	13.2 ± 4.4^a^	–	77.0 ± 4.2^a^	77.3 ± 4.8^a^	76.6 ± 4.9^a^	–	9.9 ± 0.6^a^	9.5 ± 1.0^a^	10.2 ± 0.6^a^	–	0.03 ± 0.02^a^	0.03 ± 0.01^a^	0.05 ± 0.03^a^	–
POPO	16.1 ± 0.6^a^	16.3 ± 0.3^a^	17.2 ± 1.3^a^	–	71.7 ± 0.6^a^	71.6 ± 0.4^a^	69.6 ± 1.5^a^	–	12.2 ± 0.7^a^	12.1 ± 0.4^a^	13.1 ± 1.2^a^	–	0.01 ± 0.01^a^	0.06 ± 0.05^a^	0.07 ± 0.05^a^	–
MILO	24.0 ± 0.7^a^	24.2 ± 0.5^a^	24.2 ± 0.6^a^	–	56.9 ± 1.8^a^	56.3 ± 1.6^a^	56.4 ± 1.8^a^	–	19.0 ± 1.5^a^	19.4 ± 1.4^a^	19.3 ± 1.6^a^	–	0.02 ± 0.01^a^	0.03 ± 0.01^a^	0.05 ± 0.02^a^	

^a^The values in the same row that share the same superscript letter are not significantly different

^b^Weight percent of total fatty acids, mean and standard deviations (SD) values obtained from analyses of brand set of one oil type

**Table 5:** Significant correlations between DPPH values, PUFA/MUFA ratio in oils and oxidative stability parameters,  % of change of these parameters and fatty acid contents as well as TFA contents during storage

Months of storage	Parameter	DPPH assay in oil	DPPH assay in LF	DPPH assay in HF	PUFA/MUFA ratio
0	AV	0.422 (*p* = 0.0054)	0.317 (*p* = 0.0410)	0.523 (*p* = 0.0004)	0.387 (*p* = 0.0125)
	PV	−0.378 (*p* = 0.0137)	−0.339 (*p* = 0.0281)	0.293 (*p* = 0.0492)	−0.312 (*p* = 0.0473)
3	AV	0.408 (*p* = 0.0073)	0.295 (*p* = 0.0474)	0.553 (*p* = 0.0001)	0.3770 (*p* = 0.0172)
	% of AV^a^	–	−0.191 (*p* = 0.0259)	–	–
	PV	−0.338 (*p* = 0.0285)	−0.266 (*p* = 0.0411)	–	−0.367 (*p* = 0.0182)
	*p*AV	–	–	0.265 (*p* = 0.0478)	–
	% of CT^a^	–	–	−0.297 (*p* = 0.0461)	–
	% of MUFA^a^	–	–	–	0.403 (*p* = 0.0089)
	% of PUFA	–	–	–	−0.262 (*p* = 0.0374)
6	AV	0.428 (*p* = 0.0047)	0.323 (*p* = 0.0367)	0.540 (*p* = 0.0002)	0.405 (*p* = 0.0086)
	PV	−0.309 (*p* = 0.0463)	–	–	–
	% of PV^a^	–	0.251 (*p* = 0.0383)	–	–
	% of CD^a^	0.349 (*p* = 0.0235)	0.322 (*p* = 0.0382)	–	0.279 (*p* = 0.0377)
	% of CT^a^	–	−0.268 (*p* = 0.0358)	–	–
	% of SFA^a^	–	–	–	0.366 (*p* = 0.0147)
	TFA	−0.261 (*p* = 0.0365)	−0.224 (*p* = 0.0464)	−0.423 (*p* = 0.0357)	–
12	AV	–	–	–	0.687 (*p* = 0.0023)
	% of AV^a^	−0.343 (*p* = 0.0407)	−0.358 (*p* = 0.0323)	−0.325 (*p* = 0.0374)	–
	*p*AV	−0.332 (*p* = 0.0212)	–	–	–
	CD	–	–	–	0.500 (*p* = 0.0345)
	CT	–	–	–	0.662 (*p* = 0.0028)
	% of CT^a^	–	–	−0.427 (*p* = 0.0281)	–
	% of PUFA^a^	−0.548 (*p* = 0.0316)	−0.483 (*p* = 0.0368)	–	−0.515 (*p* = 0.0343)

In the Table, are presented *R* and *p* values obtained using the Spearman rank correlation test

^a^% of change compared to fresh oil

### Oxidative Stability Parameters of Oils

The acid value (AV) measures the content of free fatty acids formed upon the hydrolytic degradation of lipid molecules, thus contributing to the reduction of the shelf life of the oil [5]. The AV of fresh and stored oils are shown in Table 6. The acid value of each of these cold-pressed oils in each of the indicated periods of storage was within the limit of up to 4 mg KOH/g of oil, according to the Codex Alimentarius Commission standard for cold-pressed and virgin oils [32]. Gorjanović et al. [33] found considerably higher concentrations of acids in the fresh samples of PUMO, as high as 1.75 mg KOH/g. Other authors, too, reported higher levels of this parameter in SESO, WALO, LINO, FLAO and SAFO [22, 34, 35] in comparison with our results. Assessment of the relationship between the antiradical activity of fresh oils and oxidative stability parameters as measured during the shelf life of oils showed a statistically significant positive correlation of AV values in fresh oils with DPPH values in nonfractionated and fractionated oils (Table 5). These effects were constant for 6 months of storage. However, a significant negative correlation between antiradical activity of the lipophilic fraction of oil and percentage of AV change after 3 months of storage suggests that lipophilic antioxidants decelerate aldehyde formation in oils. It was observed both for lipophilic and hydrophilic fractions of oils, and also in nonfractionated oil after 12 months of storage. In non-refined cold-pressed oils, adverse relations were observed between acidity and DDPH values [36]. However, the studied oils could have been subjected to refining methods commercially used in manufacturing cold-pressed oils, such as deacidification. This could result in a decrease in AV as well as antioxidant contents, so this process could influence the observed relationship [37].

**Table 6:** The acid values (AV) in fresh and stored oils (mg KOH/g)

Oil type	Mean value ± SD^A^ (range)				Percentage of mean change after whole period of shelf life^B^ (range)
	Months of storage				
	0	3	6	12	
MACO	0.08 ± 0.04 (0.04–0.11)^a^	0.08 ± 0.04 (0.04–0.11)^a^	0.10 ± 0.04 (0.06–0.13)^a^	0.08 ± 0.05 (0.04–0.13)^a^	2 (−64–73)
AVO	0.09 ± 0.02 (0.07–0.11)^a^	0.11 ± 0.03 (0.09–0.14)^a^	0.12 ± 0.03 (0.10–0.15)^a^	0.15 ± 0.08 (0.11–0.20)^a^	55 (46–82)
SESO	0.11 ± 0.06 (0.02–0.20)^a^	0.11 ± 0.08 (0.02–0.21)^a^	0.12 ± 0.07 (0.04–0.22)^a^	0.09 ± 0.05 (0.05–0.11)^a^	−23 (−50–119)
SAFO	0.18 ± 0.04 (0.14–0.23)^a^	0.17 ± 0.02 (0.14–0.18)^a^	0.21 ± 0.03 (0.18–0.24)^a^	0.18 ± 0.09 (0.14–0.21)^a^	1 (−13–33)
PUMO	0.26 ± 0.15 (0.14–0.46)^a^	0.21 ± 0.09 (0.16–0.31)^a^	0.24 ± 0.07 (0.19–0.32)^a^	0.69 ± 0.29 (0.20–1.54)^a^	164 (10–1,000)
ROSO	0.09 ± 0.02 (0.07–0.11)^a^	0.10 ± 0.03 (0.07–0.12)^a^	0.12 ± 0.02 (0.11–0.15)^a^	–	38 (10–44)
LINO	0.09 ± 0.04 (0.08–0.17)^a^	0.11 ± 0.02 (0.09–0.16)^ab^	0.13 ± 0.02 (0.11–0.17)^b^	–	44 (0–71)
FLAO	0.17 ± 0.07 (0.08–0.26)^a^	0.17 ± 0.12 (0.09–0.25)^a^	0.19 ± 0.14 (0.09–0.29)^a^	–	14 (13–14)
WALO	0.20 ± 0.13 (0.04–0.32)^a^	0.18 ± 0.15 (0.04–0.34)^a^	0.20 ± 0.12 (0.05–0.34)^a^	–	−3 (−6–36)
HEMO	0.24 ± 0.13 (0.10–0.39)^a^	0.23 ± 0.16 (0.10–0.41)^a^	0.25 ± 0.15 (0.13–0.42)^a^	–	8 (3–40)
POPO	0.43 ± 0.26 (0.17–0.76)^a^	0.46 ± 0.29 (0.16–0.74)^a^	0.61 ± 0.37 (0.21–0.94)^a^	–	41 (22–37)
MILO	0.69 ± 0.33 (0.44–1.06)^a^	0.68 ± 0.29 (0.45–1.01)^a^	0.71 ± 0.31 (0.41–1.06)^a^	–	4 (0–9)

The values in the same row that do not share the same superscript letter are significantly different

^A^Mean and standard deviations (SD) values obtained from analyses of brand set of one oil type

^B^% of mean change compared to fresh oil

PV defines the content of lipid hydroperoxides in oils formed under conditions of auto- and photo-oxidation. All the oils under study (fresh and stored) were characterized by low mean values of PV (Table 7), and none of them exceeded the recommended limit for cold-pressed oils of 15 mequiv O_2_/kg [32]. In the majority of the tested oils, increased PV value was observed after 3, 6, and 12 months, but a statistically significant difference was reported only in SESO (up to 191 %) and POPO (up to 145 %) at the end of its shelf life. A wide range of PV in fresh AVO brands was noted (4.42–15.74); moreover in two individual samples of AVO, this parameter was slightly above the recommended level. Nevertheless, the level of hydroperoxides remained unchanged throughout the whole period of AVO storage. The data published so far regarding PV in the oils covered by this study, are scarce and only limited to oils that were not subjected to storage. Gorjanović et al. [33] reported the PV for PUMO pressed from three Cucurbita pepo varieties was at a lower range (3.44–5.54 mequiv O_2_/kg), while Wroniak et al. [29] and Czaplicki et al. [35] quote values for SESO, FLAO, SAFO and WALO 1.5—three times higher than those presented in this work. In our study, a linear decrease of PV values in nonfractionated and fractionated oils was observed in fresh oils, and also after 3 months of storage, as the DPPH value increased in nonfractionated and fractionated oils (Table 5). Longer shelf life did not result in this correlation in fractionated oils, moreover after 6 months of storage, higher DPPH values in the lipophilic fractions were accompanied by an increase in percentage of PV. No correlation was observed in hydrophilic fractions in either fresh or stored oils. So the use of DPPH protocol to predict hydroperoxide formation is limited to the lipophilic fractions of oils and cannot be a hallmark of oxidative resistance during longer shelf life.

**Table 7:** The peroxide values (PV) in fresh and stored oils (mequiv O_2_/kg)

Oil type	Mean value ± SD^A^(range)				Percentage of mean change after whole period of shelf life^B^ (range)
	Months of storage				
	0	3	6	12	
MACO	2.46 ± 1.21 (1.67–3.86)^a^	3.41 ± 1.77 (2.15–5.43)^a^	5.20 ± 4.02 (2.75–9.85)^a^	5.87 ± 3.33 (3.81–9.71)^a^	138 (119–152)
AVO	9.55 ± 5.35 (4.42–15.74)^a^	9.99 ± 5.76 (3.87–15.30)^a^	7.60 ± 3.06 (5.17–11.04)^a^	10.99 ± 3.54 (7.67–14.72)^a^	15 (−14–74)
SESO	1.42 ± 0.69 (0.70–2.20)^a^	2.32 ± 0.41 (2.01–2.90)^ab^	3.06 ± 1.06 (1.99–4.08)^ab^	4.13 ± 0.94 (3.13–5.01)^b^	191 (60–375)
SAFO	4.20 ± 1.86 (2.26–6.27)^a^	5.03 ± 2.04 (3.01–7.26)^a^	6.69 ± 3.41 (3.44–11.27)^a^	9.65 ± 3.96 (5.88–14.54)^a^	130 (113–160)
PUMO	6.04 ± 3.03 (2.10–9.43)^a^	6.97 ± 5.32 (2.72–12.94)^a^	7.24 ± 4.68 (3.38–12.45)^a^	7.39 ± 3.87 (3.10–10.63)^a^	22 (−48–58)
ROSO	2.97 ± 0.87 (2.02–4.00)^a^	2.80 ± 0.86 (2.25–3.78)^a^	4.54 ± 0.96 (3.93–5.64)^a^	–	53 (1–122)
LINO	1.12 ± 0.94 (0.27–2.80)^a^	1.26 ± 0.64 (0.62–2.30)^a^	1.96 ± 0.92 (0.37–2.80)^a^	–	74 (−20–7,056)
FLAO	0.60 ± 0.10 (0.50–0.69)^a^	0.48 ± 0.23 (0.21–0.64)^a^	0.34 ± 0.18 (0.22–0.47)^a^	–	−43 (−57–(-32))
WALO	2.17 ± 0.49 (1.69–2.79)^a^	4.30 ± 2.43 (2.64–7.09)^a^	4.70 ± 2.11 (2.81–6.97)^a^	–	116 (66–150)
HEMO	3.23 ± 0.90 (2.63–4.55)^a^	4.32 ± 1.91 (2.75–6.45)^a^	8.66 ± 6.39 (4.91–16.04)^a^	–	168 (11–426)
POPO	2.77 ± 1.20 (1.41–3.68)^a^	4.21 ± 1.30 (2.72–5.08)^a^	6.80 ± 0.70 (6.27–7.59)^a^	–	145 (78–438)
MILO	3.15 ± 2.20 (1.04–5.43)^a^	3.43 ± 3.08 (1.44–6.98)^a^	4.77 ± 3.51 (1.90–8.68)^a^	–	51 (−31–735)

The values in the same row that do not share the same superscript letter are significantly different

^A^Mean and standard deviations (SD) values obtained from analyses of brand set of one oil type

^B^% of mean change compared to fresh oil

The *p*-AV reflects the content of secondary products of lipid oxidation, resulting from the decomposition of hydro-peroxides. *p*-AV along with PV may therefore offer elucidation of the rancidity of oils [38]. The lowest *p*-AV of fresh oils was found in all SESO brands (range 0.2–0.3) and the values did not change during storage (Table 8). The largest variability of *p*-AV in fresh oils occurred between brands of ROSO (4.22–11.88) and PUMO (1.48–8.17). The highest peak was found in ROSO, with an average 68 % increase in the sixth months of storage. Nevertheless, this increment was not statistically significant. The health safety of oils in relation to *p*-AV is difficult to assess because of the lack of an established limit of this parameter in cold-pressed oils. In reviewing the literature, the data on the *p*-AV of the oils analyzed in this work were very limited. The results of *p*-AV determination in commercially available cold-pressed oils obtained by Wroniak et al. [29] showed lower values for SAFO (0.23), LINO (0.36) and FLAO (0.48), and higher ones in PUMO (6.92) and WALO (6.07) than those found in our study. A significant negative correlation between DPPH values in nonfractionated oils under study and *p*-AV of the stored oils was shown after 12 months of shelf life, which indicates that the secondary oxidative product formation in cold-pressed oils rich in antioxidants is very slow (Table 5) [9].

**Table 8:** The *p*-anisidine values (*p*-AV) in fresh and stored oils

Oil type	Mean value ± SD^A^ (range)				Percentage of mean change after whole period of shelf life^B^ (range)
	Months of storage				
	0	3	6	12	
MACO	2.85 ± 0.57 (2.44–3.50)^a^	2.86 ± 0.73 (2.36–3.70)^a^	3.01 ± 0.95 (2.36–4.10)^a^	3.11 ± 1.21 (2.31 – 4.50)^a^	9 (–5–29)
AVO	7.15 ± 1.39 (5.83–8.90)^a^	8.50 ± 3.74 (6.28–12.82)^a^	7.83 ± 2.14 (6.51–10.29)^a^	8.47 ± 1.97 (6.96 – 10.70)^a^	18 (11–40)
SESO	0.25 ± 0.05 (0.20–0.30)^a^	0.22 ± 0.04 (0.25–0.33)^a^	0.28 ± 0.07 (0.21–0.34)^a^	0.37 ± 0.06 (0.31 – 0.43)^a^	47 (22–115)
SAFO	2.83 ± 2.27 (0.53–5.80)^a^	2.18 ± 1.83 (0.61–3.49)^a^	2.25 ± 1.92 (0.54–3.93)^a^	2.70 ± 2.29 (0.71 – 5.20)^a^	−5 (−12–46)
PUMO	4.83 ± 3.35 (1.48–8.17)^a^	5.44 ± 2.81 (0.55–8.15)^a^	6.15 ± 3.62 (2.44–9.67)^a^	6.41 ± 3.69 (2.63 – 10.00)^a^	33 (22–78)
ROSO	8.60 ± 3.20 (4.20–11.88)^a^	12.33 ± 1.96 (10.28 –14.19)^a^	14.44 ± 2.73 (11.77–17.22)^a^	–	68 (21–91)
LINO	1.07 ± 0.49 (0.60–1.80)^a^	1.16 ± 0.67 (0.58–2.30)^a^	1.51 ± 0.97 (0.67–2.60)^a^	–	41 (−12–155)
FLAO	0.87 ± 0.57 (0.53–1.72)^a^	0.90 ± 0.56 (0.49–1.54)^a^	1.09 ± 0.73 (0.64–1.93)^a^	–	26 (12–35)
WALO	2.57 ± 0.79 (1.87–3.30)^a^	3.57 ± 0.51 (3.00–4.00)^a^	3.81 ± 0.57 (3.20–4.33)^a^	–	48 (31–71)
HEMO	2.74 ± 1.48 (1.80–4.96)^a^	3.47 ± 2.13 (2.17–5.93)^a^	3.66 ± 2.53 (2.14–6.58)^a^	–	33 (2–40)
POPO	0.75 ± 0.20 (0.49–0.96)^a^	0.81 ± 0.38 (0.48–1.22)^a^	1.01 ± 0.38 (0.67–1.41)^a^	–	34 (23–47)
MILO	2.56 ± 1.98 (3.42–4.69)^a^	3.42 ± 2.11 (3.63–5.13)^a^	3.63 ± 2.32 (1.08–5.61)^a^	–	42 (0–52)

^a^The values in the same row that share the same superscript letter are not significantly different

^A^Mean and standard deviations (SD) values obtained from analyses of brand set of one oil type

^B^% of mean change compared to fresh oil

The formation of hydroperoxides from PUFA in the early stages of oxidation may result in double bond isomerization. The determination of conjugated dienoic and trienoic fatty acid derivatives (CD and CT) enable definition of the oxidation state of an oil, in addition to PV and *p*-AV [38].

The CD content in fresh oils ranged from 1.86 (% E) in MACO up to 4.31 (% E) in PUMO (Table 9). The lowest average CD content of MACO is related to its specific fatty acid composition, which is meager in linoleic acid. In three oil types rich in linoleic acid, POPO, MILO and LINO, the CD content increased significantly after 6 months of storage, (mean increase up to 86 %). Wagner et al. [39] found that a rapid increase in CD generation in POPO stored for 6 days at 40 °C was the result of mechanical damage to the poppy seeds used for the oil production. This effect was also accompanied by a considerable increase of *p*-AV.

**Table 9:** The conjugated diene (CD) and conjugated triene (CT) content in fresh and stored oils (%E)

Oil type	CD					CT				
	Mean value ± SD^A^ (range)				Percentage of mean change after whole period of shelf life^B^ (range)	Mean value ± SD^A^ (range)				Percentage of mean change after whole period of shelf life^B^ (range)
	Months of storage					Months of storage				
	0	3	6	12		0	3	6	12	
MACO	1.86 ± 0.36 (1.58–2.26)^a^	2.06 ± 0.47 (1.75–2.60)^a^	2.33 ± 0.47 (1.99–2.13)^a^	2.10 ± 1.14 (1.32–3.41)^a^	13 (−16- 51)	0.18 ± 0.04 (0.15–0.23)^a^	0.43 ± 0.12 (0.30–0.54)^a^	0.42 ± 0.30 (0.11–0.71)^a^	0.31 ± 0.09 (0.20–0.38)^a^	69 (27–149)
AVO	3.38 ± 0.82 (2.84–4.32)^a^	3.15 ± 1.00 (2.20–4.19)^a^	4.14 ± 1.52 (3.02–5.87)^a^	4.03 ± 1.01 (2.90–4.86)^a^	20 (−2–54)	0.75 ± 0.49 (0.42–1.31)^a^	0.80 ± 0.70 (0.19–1.56)^a^	1.03 ± 0.68 (0.36–1.73)^a^	0.66 ± 0.41 (0.30–1.11)^a^	−13 (−43–33)
SESO	3.48 ± 0.50 (3.04–4.18)^a^	3.48 ± 0.64 (2.82–4.36)^a^	3.85 ± 0.49 (3.46–4.57)^a^	3.48 ± 0.16 (3.32–3.65)^a^	0 (−13–19)	0.96 ± 0.55 (0.38–1.46)^a^	0.79 ± 0.55 (0.39–1.60)^a^	0.81 ± 0.50 (0.46–1.56)^a^	0.68 ± 0.53 (0.45–1.19)^a^	−29 (−67–58)
SAFO	3.20 ± 1.21 (1.98–4.35)^a^	3.65 ± 1.70 (2.01–5.27)^a^	4.50 ± 1.62 (2.73–6.22)^a^	4.40 ± 1.93 (2.71–6.26)^a^	38 (19–52)	0.65 ± 0.46 (0.19–1.05)^a^	0.72 ± 0.61 (0.18–1.34)^a^	0.82 ± 0.55 (0.30–1.44)^a^	0.68 ± 0.53 (0.18–1.22)^a^	6 (−17–16)
PUMO	4.31 ± 1.02 (3.30–5.35)^a^	4.37 ± 0.60 (3.98–5.06)^a^	4.37 ± 0.31 (4.17–4.73)^a^	4.24 ± 0.19 (4.06–4.44)^a^	−2 (−24–34)	2.96 ± 0.51 (2.57–3.53)^a^	2.93 ± 0.68 (2.27–3.62)^a^	2.91 ± 0.52 (2.34–3.36)^a^	2.47 ± 0.11 (2.36–2.60)^a^	−16 [−26–(−7.04)]
ROSO	2.88 ± 0.47 (2.35–3.21)^a^	3.31 ± 0.30 (3.01–3.60)^a^	5.70 ± 2.63 (4.02–8.74)^a^	–	98 (25–272)	0.61 ± 0.45 (4.02–8.74)^a^	0.91 ± 0.16 (0.81–1.09)^a^	1.31 ± 0.00 (1.30–1.31)^a^	–	113 (28–906)
LINO	1.92 ± 0.14 (1.75–2.13)^a^	2.08 ± 0.40 (1.49–2.62)^ab^	2.53 ± 0.26 (2.13–2.90)^b^	–	31.5 (5.6–57)	0.25 ± 0.11 (0.14–0.37)^a^	0.26 ± 0.07 (0.13–0.34)^a^	0.40 ± 0.21 (0.21–0.74)^a^	–	64 (−43–280)
FLAO	1.94 ± 0.28 (1.68–2.23)^a^	1.92 ± 0.07 (1.87–1.99)^a^	2.44 ± 0.26 (2.14–2.63)^a^	–	26 (18–33)	0.30 ± 0.27 (0.09–0.61)^a^	0.36 ± 0.14 (0.24–0.52)^a^	0.43 ± 0.15 (0.28–0.57)^a^	–	44 (−6–200)
WALO	3.50 ± 2.98 (1.37–6.90)^a^	3.53 ± 3.02 (1.78–7.02)^a^	4.52 ± 2.93 (1.87–7.63)^a^	–	29 (−19–202)	0.79 ± 0.84 (0.20–1.75)^a^	0.8 ± 0.85 (0.21–1.78)^a^	1.68 ± 1.46 (0.11–3.01)^a^	–	112 (−73–1,411)
HEMO	3.22 ± 1.69 (2.04–5.16)^a^	3.03 ± 1.32 (2.06–4.53)^a^	2.99 ± 0.86 (2.28–3.95)^a^	–	−7 (−24–12)	0.50 ± 0.19 (0.37–0.72)^a^	0.40 ± 0.08 (0.31–0.46)^a^	0.59 ± 0.14 (0.62–0.71)^a^	–	18 (−14–71)
POPO	2.28 ± 0.81 (1.62–3.18)^a^	2.17 ± 0.43 (1.88–2.66)^ab^	4.26 ± 0.27 (9.05–11.39)^b^	–	86 (69–102)	0.18 ± 0.08 (0.13–0.27)^a^	0.33 ± 0.15 (0.22–0.50)^a^	0.67 ± 0.48 (0.11–0.96)^a^	–	278 (−12–639)
MILO	2.62 ± 0.31 (2.34–2.95)^a^	2.83 ± 0.37 (2.41–3.05)^ab^	3.92 ± 0.23 (3.69–4.11)^b^	–	41 (28–49)	0.48 ± 0.16 (0.30–0.60)^a^	0.51 ± 0.15 (0.35–0.64)^a^	0.52 ± 0.1 (0.39–0.59)^a^	–	9 (−1–29)

The values in the same row that do not share the same superscript letter are significantly different

^A^Mean and standard deviations (SD) values obtained from analyses of brand set of one oil type

^B^% of mean change compared to fresh oil

The CT content of fresh oils ranged from 0.18 to 2.96 % E (Table 7). MACO and POPO (both 0.18 % E) were characterized by the lowest CT level, undoubtedly related to a scarce concentration of α-linolenic acid [24, 28]. However, fresh PUMO, with low levels of this fatty acid (0.3 %), was characterized by the highest average CT value (2.96 % E, equivalent to ca. 0.3 % CT in total fatty acids) indicating that the high CT value of PUMO cannot simply be attributed to α-linolenic acid oxidation [40]. The study shows rather constant CT values of oils, with no signs that high-linolenic oils have a higher susceptibility to isomerization during the whole period of their shelf life. Moreover the correlations between DPPH values and percentage changes in the CD and CT of the studied oils could suggest that antioxidants prevent the formation of conjugated trienoic acid and do not inhibit diene isomerization throughout the whole period of storage (Table 5).

### PUFA/MUFA ratio and oil oxidative stability

As the oils rich in PUFA were found to have high antiradical activity, a similar relationship was observed between the PUFA/MUFA ratio and oxidative stability parameters as in the case of DPPH values (Table 5).

## Conclusions

In conclusion, the measured radical scavenging activity of the studied cold-pressed oils varied from 0.17 to 2.32 mM TEAC/kg. However, the oxidative stability of oils during their shelf life did not simply reflect their antioxidant potential. AVO and POPO were characterized by low total antioxidant activity, and these oils showed clear signs of deterioration: there was high *p*-AV and PV in fresh AVO, and a significant increase in PV and CD content during storage indicates PUFA oxidation in POPO. On the other hand, low values of measured oxidation parameters during the whole period of storage were found in MACO, which exhibited very low antioxidant activity, while considerable amounts of secondary products of lipid oxidation were determined in ROSO, reflecting the relatively advanced process of its oxidation, despite the high antioxidant activity of this oil. Through the assessment of the relationship between antiradical activity and the oxidative stability of oils, it can be proposed that a DPPH assay predicts the formation of primary (PV, CT) and secondary (*p*-AV) oxidation products in cold-pressed oils, however the correlations differ in fractionated and nonfractionated oils.
